# Unravelling the mechanism by which vildagliptin and linagliptin inhibit pyroptosis in lung injury through the NLRP3 inflammatory pathway in type 1 diabetic rats

**DOI:** 10.1038/s41598-025-07204-1

**Published:** 2025-06-25

**Authors:** Ahmed A. Sedik, Nesma M. E. Abo El-Nasr, Wagdy K. B. Khalil, Aliaa E. M. K. El-Mosallamy

**Affiliations:** 1 Pharmacology Department, Medical Research and Clinical Studies Institute, National Research Centre, El- Buhouth St., Dokki, Cairo 12622 Egypt; 2 Department of Cell Biology, Biotechnology Research Institute, National Research Centre, El- Buhouth St., Dokki, Cairo 12622 Egypt

**Keywords:** Diabetes mellitus, Streptozotocin, Lung injury, Pyroptosis, Vildagliptin, Linagliptin, Biochemistry, Diseases

## Abstract

Diabetes mellitus (DM) represents a multifactorial condition linked to hyperglycemia, which, can lead to damage across multiple organs, including the lungs. Nod-like receptor protein-3 (NLRP3)- mediated pyroptosis could contribute to the onset of DM consequences. Several approaches have been established aimed to minimizing the complications associated with DM. Among these, linagliptin and vildagliptin, di-peptidyl peptidase-4 (DPP-4) inhibitors, are known to exert not only antihyperglycemic effects but also additional beneficial biological activities. The current study investigated the impact of linagliptin and vildagliptin on pulmonary function, oxidative stress, and NLRP3-induced pyroptosis in rats. Thirty-two male Sprague Dawley rats were given a 7-day acclimatization period. A single intraperitoneal injection of freshly produced STZ (60 mg/kg) was utilized to develop DM type-1 in rats. Following STZ treatment, all rats were given a 5% glucose solution overnight. Blood glucose levels were monitored in overnight fasted rats 72 h later, with a threshold of 250 mg/dL or higher confirming the onset of DM. The diabetic rats were randomly allocated to treated daily with either vildagliptin (5 mg/kg/p.o.) or linagliptin (5 mg/kg/p.o.) for 30 days. Additionally, the typical control group received merely the vehicle. The findings revealed that vildagliptin improves pulmonary dysfunctions associated with DM by restoring glucose homeostasis, insulin, redox marker levels, and inflammatory indices. Additionally, the NLRP3-pyroptosis-mediated IL-1β was suppressed. Vildagliptin has been shown to mitigate the detrimental effects of diabetes mellitus (DM) on the lungs, as evidenced by a reduction in pathological lung alterations and a decrease in Caspase 3 expression, which is indicative of immunohistochemical changes. In conclusion, pyroptosis triggered by the NLRP3 inflammasome possibly exacerbate diabetic pulmonary injury in rats. Vildagliptin is superior to linagliptin in ameliorating diabetes-induced lung injury primarily via targeting the NLRP3 inflammasome pathway.

## Introduction

Diabetes mellitus (DM) is a lifelong metabolic condition that is expected to impact approximately 642 million people globally by 2040^1^. In developed countries, Diabetes is the third leading cause of death, following cardiovascular diseases and cancer, possibly caused by a shortage of insulin secretion, resistance to insulin’s peripheral actions, or both^2^. DM is distinguished by chronic hyperglycemia that can affect the brain, kidney, liver, heart, eyes, and skeletal muscle^3^. Diabetes affects several organs, including the lungs^4^where it has been shown to affect pneumocytes (type II) that impair surfactant production and secretion, resulting in diminished elasticity as well as lung volume^5^. Despite these findings, few studies have focused on the pathophysiological processes by which diabetes impacts pulmonary function.

Pyroptosis considered as an inflammatory process of programmed cell death, acting as an essential part of the innate immune response. While it plays a protective role in defending multicellular organisms against pathogens, its overactivation can cause chronic inflammatory phase^6^. Central to the execution of pyroptosis are inflammasomes, large multi-protein complexes that facilitate the activation of pro-inflammatory cytokines, thereby modulating innate immune system’s response^7^. Markedly, the inflammasome NLRP3 (Nod-like receptor pyrin domain containing 3) plays a crucial role in the response of innate immune. Under hyperglycemic conditions, reactive oxygen species (ROS) trigger NLRP3 in pancreatic β cells, which in turn induces the caspase-1-dependent production of interleukin-1β (IL-1β). This inflammatory cascade results in aberrant insulin secretion from β cells, thereby contributing to the progression of diabetes. Therefore, the activation of the NLRP3 inflammasome is closely associated with pyroptosis in the pathogenesis of diabetes^8^. Gasdermin D (GSDMD), known as a biological precursor that participates in the traditional inflammasome pathway, which causes pyroptosis. GSDMD promotes the development of IL-1β production and produces cytotoxic holes in the cell membrane. Here, it is simultaneously cleaved by both pro-caspase-1 and caspase-1 (Cas-1), and drawn to the inflammasome NLRP3^9^.

Emerging therapeutic strategies for diabetes encompass islet transplantation, gene therapy, advanced the insulin analogues and delivery systems, and the hunt for new medicines to prevent and/or stimulate islet β-cell proliferation and regeneration^10^. In this regard, gastrointestinal hormones such as glucose-dependent insulinotropic polypeptide (GIP) and glucagon-like peptide-1 (GLP-1), known as incretins, enhance glucose-stimulated insulin secretion. These hormones not only stimulate insulin biosynthesis but also promote β-cell proliferation while inhibiting apoptosis^11^.

Dipeptidyl peptidase-4 (DPP-4) inhibitors are antidiabetic medications that block the degradation of incretin hormones, such as GIP and GLP-1, thereby enhancing insulin secretion and improving glycemic control^12^. Beyond their metabolic effects, DPP-4 is also highly expressed on endothelial and epithelial cells in lung tissues, prompting interest in the use of DPP-4 inhibitors in various pulmonary conditions, including pulmonary fibrosis, asthma, acute lung injury, and even COVID-19 ^13,14^. Moreover, DPP-4 inhibitors have been shown to delay or prevent hyperglycemia in diabetic animal models^15^although the precise mechanisms remain not fully understood.

Among DPP-4 inhibitors, vildagliptin and linagliptin have demonstrated potential in alleviating lung injury, especially pulmonary fibrosis, including improving pulmonary microvascular barrier integrity and reducing inflammation^16^ However, while both agents have shown protective effects in lung disease models, no prior study has directly compared the effects of vildagliptin and linagliptin on pyroptosis, particularly the NLRP3 inflammasome-mediated pyroptosis pathway, in the context of lung injury associated with diabetes. This represents a significant gap in the literature, given the emerging role of pyroptosis in mediating inflammation and tissue damage in diabetes-related complications^17^. Hence, the current study was designed to evaluate and compare the impact of vildagliptin and linagliptin on pulmonary function via NLRP3-mediated pyroptosis in STZ-induced diabetic male Sprague-Dawley rats, to clarify their potential mechanisms of action in diabetic lung injury.

## Materials and methods

### Animals

Adult male Sprague- Dawley rats weighing between 200 and 220 g, were obtained from the Animal house of the National Research Centre in Dokki, Giza. The rats were housed in cages at room temperature with adequate ventilation and given water and a commissural rat chow diet. They were given two weeks to acclimate before beginning the trial. The study was authorized by the National Research Centre’s Medical Research Ethics Committee (MREC) under authorization number (130601102). All methods were performed in accordance with the relevant guidelines and regulations. The present study is reported in accordance with ARRIVE guidelines.

### Chemicals

STZ was purchased in vial containing 100 mg of STZ powder from Sigma‒Aldrich, USA. STZ prepared in 0.1 M citrate buffer at pH 4.5. Vildagliptin and linagliptin were obtained from Novartis Pharma, Basel, Switzerland, and Ingelheim Pharma GmbH, Germany, respectively, and were dissolved in 0.9% (1 mg/ml) normal saline to a net concentration is 5 mg/kg.

### Experimental design

Thirty-two eight-week-old male Sprague Dawley-rats weighing between 200 and 220 g were allowed to acclimatize for 7 days. A single injection of freshly prepared STZ (60 mg/kg; i.p.) was used to induce type 1 diabetes in rats^18,19^. Following the STZ injection, all rats were provided with a 5% glucose solution overnight. Diabetes mellitus (DM) was diagnosed 72 h later by monitoring blood glucose levels following an overnight fast. Diabetes was defined as blood glucose levels of 250 mg/dL or higher. The selected diabetic rats were randomized into three groups assigned as one group of untreated STZ-diabetic rats and two groups of STZ diabetic rats that orally given vildagliptin (5 mg/kg/day)^20^ or linagliptin (5 mg/kg/day)^21^ once daily for 30 days. In addition, the normal control group received only the vehicle.

Following an overnight fast, the experimental rats were anaesthetized using ketamine (100 mg/kg) and xylazine (10 mg/kg). Blood samples were obtained from the eyes of the rats’ retroorbital plexus after being separated via centrifuged at 3000 rpm for 15 min at 4 °C (laborezentrifuger, cooling centrifuge, 2k15, Sigma, Germany) to evaluate the fasting glucose and insulin levels. Then, the animals were euthanized through cervical dislocation, and the lung tissues were excised and stored at − 80 °C for subsequent biochemical and gene expression analyses. Moreover, lung samples were fixed in 10% buffered formalin intended for histopathological and immunohistochemical evaluation of Caspase-3.

#### Evaluation of fasting glucose and insulin values

The fasting serum glucose levels were evaluated, and the results are expressed as mg/dl^22^. Additionally, the serum level of fasting insulin was evaluated and expressed as µIU/mL^23^.

### Lung homogenate preparation

The homogenization of lung tissue was carried out according to the methods of^24^. The resulting supernatant was used to determine the oxidative stress related parameters namely, the levels of malondialdehyde (MDA), superoxide dismutase (SOD), reduced glutathione (GSH) nuclear factor kappa B (NF-Kb), tumor necrosis factor-alpha (TNF-α), interleukin-1 beta (Il-1β) and the expression of the NLRP3 and GSDMD genes.

#### Evaluation of oxidant/antioxidant markers

The MDA and GSH contents in the lungs were evaluated using colorimetric kits from Bio*-*diagnostic, Egypt (CAT: MD 25 29)^25^ and (CAT: GR 25 11)^26^respectively. Furthermore, the SOD activity was ascertained using a spectrophotometric technique^27^.

#### Estimation of the levels of NF-kB and Proinflammatory cytokines in rats’ lungs

The values of NF-kB, TNF-α, and IL-1β in the lungs were quantified using ELISA kits (USCN, Wuhan, China). The collected values are provided as pg/mL (Catalogue no.: SEB824Hu, SEA133Rb, and SEA080Hu, respectively)^28^.

#### Evaluation of the expression of the NLRP3 and GSDMD genes

Lung samples were collected from control rats, STZ-treated rats, STZ-treated with Linagliptin rats and STZ-treated with Vildagliptin. The total RNA extracted from the lung tissues by using the standard TRIzol^®^ Reagent extraction method of rats (cat # 15596-026; Invitrogen, Germany).Where, the RNA was dissolved in DEPC-treated H_2_O^29,30^. To remove any DNA residues, the total RNA was treated with 1 U of RQ1 RNAse-free DNase (Invitrogen, Germany) before being resuspended in DEPC-treated water. RNA purity was assessed by the 260/280 nm ratio (ranging from 1.8 to 2.1). Additionally, the integrity of the RNA was confirmed by examining the 28 S and 18 S ribosomal RNA bands through ethidium bromide staining using formaldehyde-containing agarose gel electrophoresis^31^.

##### Reverse transcription (RT)

The cDNA was extracted from the lung samples using a Kit of RevertAidTM First Strand cDNA Synthesis (MBI Fermentas, Germany). The kit was employed to reverse transcribe the entire poly(A) + RNA extracted from the lung tissue samples into cDNA, in a total reaction volume of 20 µl. The reaction tubes containing the RT preparations were then quickly cooled in an ice chamber before being used for real-time polymerase chain reaction (RT-PCR) amplification.

##### RT-PCR reaction

The StepOne™ Real-Time PCR System from Applied Biosystems (Thermo Fisher Scientific, Waltham, MA, USA) was used to count the number of lung tissues in samples. PCRs were performed in 25 µL reaction mixtures with 12.5 µL of 1× SYBR^®^ Premix Ex TaqTM (TaKaRa, Biotech. Co. Ltd.), 0.5 µL of 0.2 µM sense primer, 0.5 µL of 0.2 µM antisense primer, 6.5 µL of distilled water, and 5 µL of cDNA template^32,33^. Table 1. provides the sequences of the specific primers used in the study. To confirm the quality of the primers, a melting curve analysis was conducted at 95.0 °C at the end of each qPCR. The relative quantification of the target gene compared to the reference gene was calculated using the 2 − ΔΔCT method, and the primer sequences used are presented in Table 1.^34^^36^.

**Table 1:** Primers sequence used for *qRT-PCR*.

Gene	Primer sequence	NCBI reference
*NLRP3*	F: CTC GCA TTG GTT CTG AGC TC R: AGT AAG GCC GGA ATT CAC CA.	NM_001191642.1
*GSDMD*	F: AAG CCG GAA GAA GAT GGT GA R: GAA TGG AGT ACG GCA AGC AG	NM_001400994.1
*GAPDH*	F: GAG ACA GCC GCA TCT TCT TG R: TGA CTG TGC CGT TGA ACT TG	XM_063285519.1

*NLRP3*, NLR family, pyrin domain containing 3; *GSDMD*, gasdermin D; glyceraldehyde-3-phosphate dehydrogenase.

### Histopathological investigations

Lung tissues from each rat were collected promptly and fixed in 10% buffered neutral formalin for 24 h. The prepared samples were dehydrated in ethanol, cleared in xylene, embedded in paraffin, and sectioned at a thickness of 5 μm. After staining with hematoxylin and eosin(H&E), then the sections were visualized microscopically^37^. To assess type II pneumocytes and interalveolar septa thickness, ten non-overlapping fields were randomly selected from 10 slides of each rat within the study groups.

### Immunohistochemical analysis of caspase-3

Immunohistochemical assessment was used for determining the cleaved caspase-3 in the lungs of various groups^38^. The paraffin-embedded lung slices were dewaxed, rehydrated in alcohol, and treated with 3% hydrogen peroxide. The sections were subsequently treated with rabbit polyclonal anti-caspase-3 antibody (ab13847; Abcam). Dia-minobenzidine was used to visualize the immunological reaction. The percentage of the overall area stained with the anti-caspase-3 antibody was measured using the Leica Quin 500 analyser computer system (Leica Microsystems, Switzerland)^39^.

### Statistical analysis

Quantitative comparisons in the study were performed using one-way ANOVA, with Tukey’s multiple comparison test applied through GraphPad Prism software version 8.0 (USA). The results are expressed as the mean ± SEM of 8 rats, and a p-value of ≤ 0.05 was considered to indicate statistical significance.

## Results

###  Effect of the administration of vildagliptin and linagliptin on the fasting glucose and insulin values in diabetic rats

**Fig. 1:**
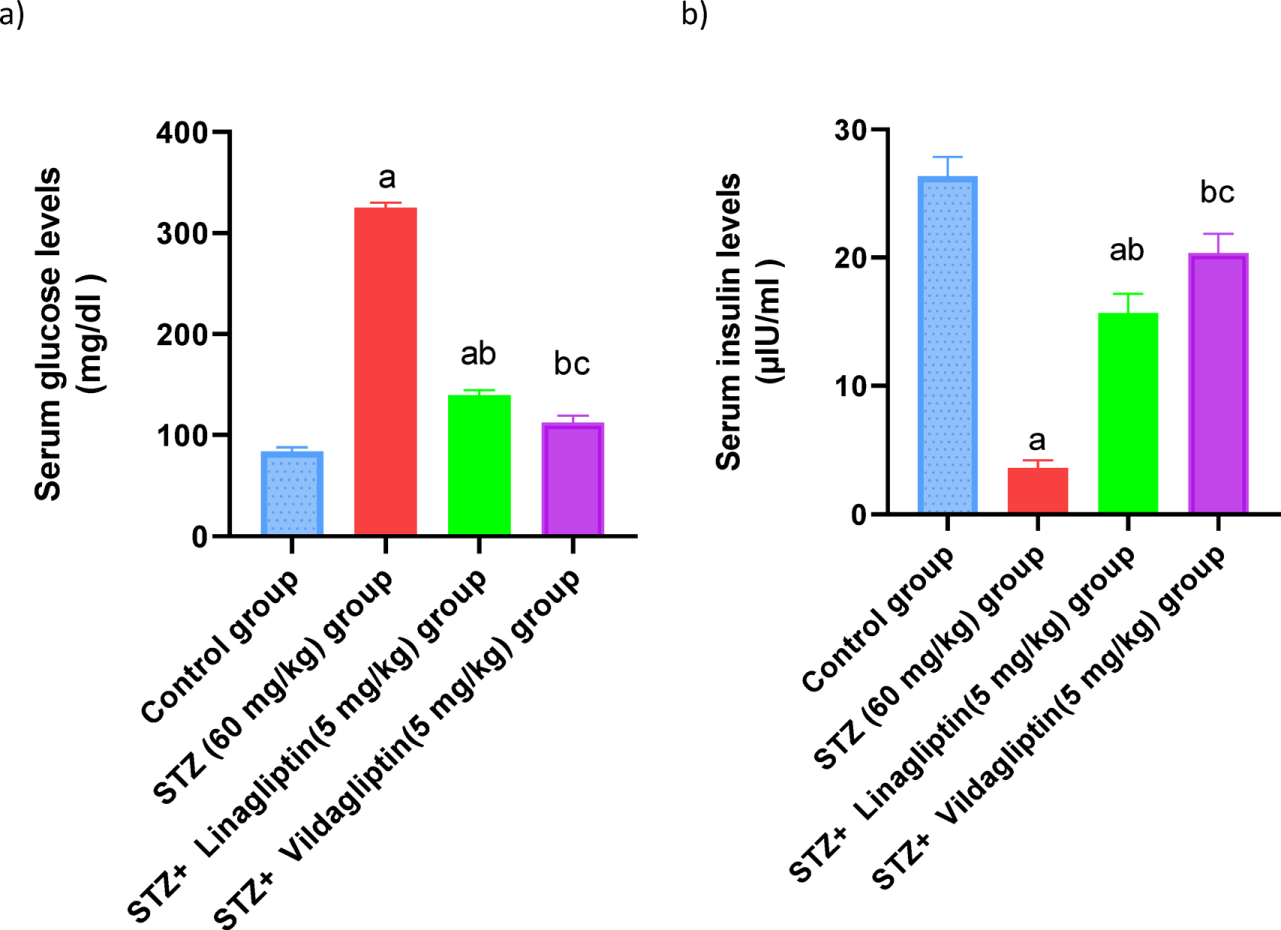
Effect of administration of linagliptin and vildagliptin on the serum glucose and insulin levels in diabetic rats. DM was induced by intraperitoneal injection of a single dose of STZ (60 mg/kg b.wt.). After DM was confirmed, selected rats were orally treated with vildagliptin (5 mg/kg/day) or linagliptin (5 mg/kg/day) once daily for 30 days. At the end of the experiment, after fasting overnight, the experimental rats were euthanized with ketamine (100 mg/kg) plus xylazine (10 mg/kg). Then, blood samples were collected from to evaluate the fasting glucose and insulin levels. Data are presented as mean ± SEM (8). a Significant difference from control group *p* < *0.05*.b Significant difference from STZ group *p* < *0.05*. c Significant difference from STZ + linagliptin(5 mg/kg) group *p* < *0.05*.

The STZ treated rats showed a significant increase of the fasting glucose levels by 3.8-fold and a decrease in insulin values by 13.5%, when compared to normal control group. However, treatment with linagliptin (5 mg/kg) and vildagliptin (5 mg/kg) resulted in a significant reduction in fasting glucose levels (*p* < 0.05) by 42.7% and 39.7%, respectively, along with a notable increase in insulin levels by 4.3-fold and 5.7-fold, compared to the STZ group. (Fig. 1).

###  Effect of the administration of vildagliptin and linagliptin on the MDA values and the activities of GSH and SOD in diabetic rats

**Fig. 2:**
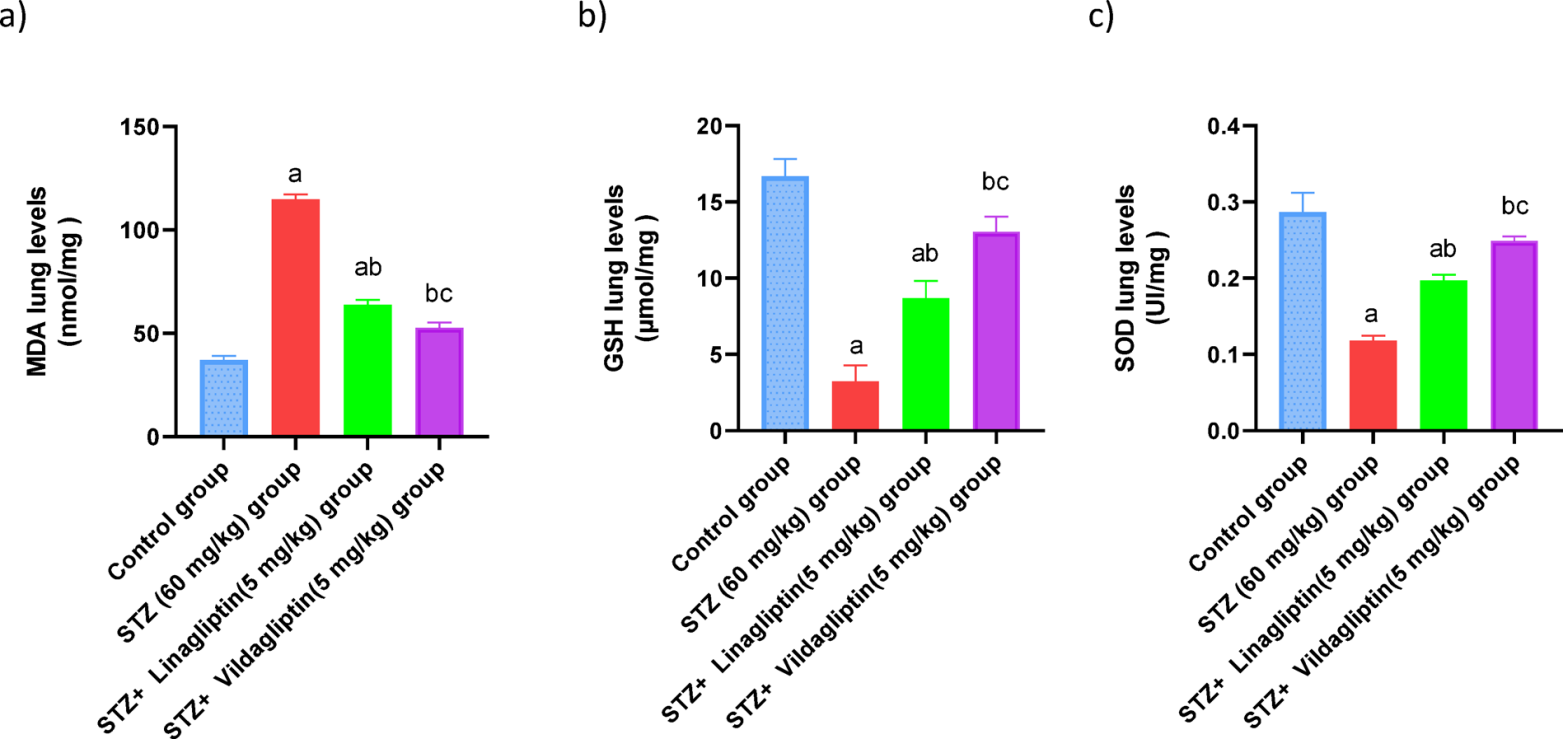
Effect of administration of linagliptin and vildagliptin on the lung values of MDA, GSH and SOD in diabetic rats. DM was induced by intraperitoneal injection of a single dose of STZ (60 mg/kg b.wt.). After DM was confirmed, selected rats were orally treated with vildagliptin (5 mg/kg/day) or linagliptin (5 mg/kg/day) once daily for 30 days. At the end of the experiment, rats were sacrificed by cervical dislocation and lungs were homogenized for evaluation of the lung values of MDA, GSH and SOD. Data are presented as mean ± SEM (8). a Significant difference from control group *p* < *0.05*.b Significant difference from STZ group *p* < *0.05*. c Significant difference from STZ + linagliptin(5 mg/kg) group *p* < *0.05*.

The STZ treated rats showed a significant elevation in MDA values by 3-fold with a marked decrease in GSH and SOD values by 19.5%, 41%, as compared to normal control group. Conversely, administration of linagliptin (5 mg/kg) revealed a decrease in the MDA values (*p* < 0.05) significantly by 55.5%. On the other hand, linagliptin showed a marked increase in GSH and SOD activities by 2.7fold, 1.6-fold, compared to the STZ group. The STZ + Vildagliptin (5 mg/kg) group revealed a significant decrement in the MDA values (*p* < 0.05) by 45% and a marked increase in GSH and SOD activities by 4.1fold, 2-fold as compared to the STZ group (Fig. 2).

###  Effect of the administration of vildagliptin and linagliptin on the levels of nuclear factor-κB (NF-κB) and proinflammatory cytokines in the lungs of diabetic rats

**Fig. 3:**
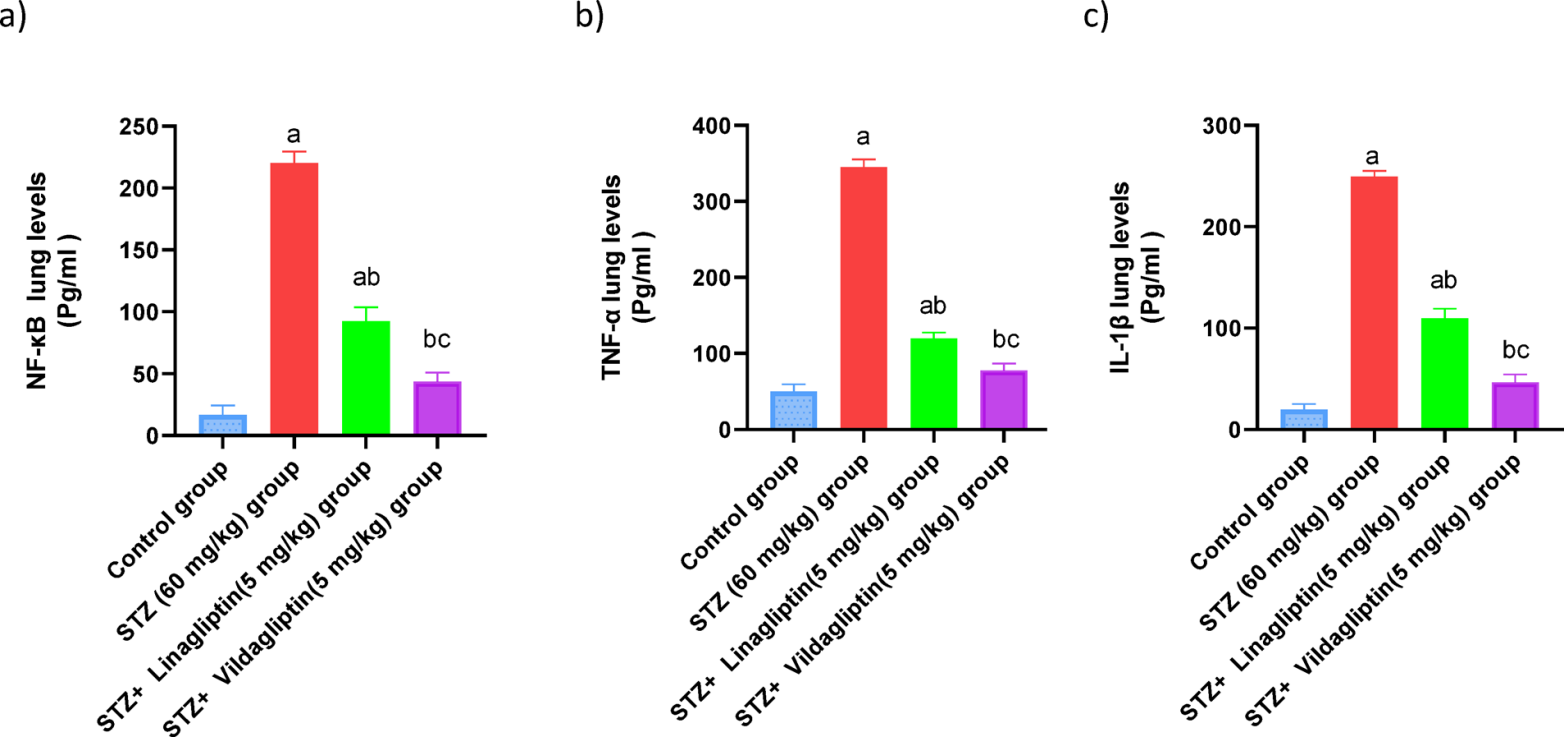
Effect of administration of linagliptin and vildagliptin on the lung values of NF-kB, TNF-α, and IL-1β in diabetic rats. DM was induced by intraperitoneal injection of a single dose of STZ (60 mg/kg b.wt.). After DM was confirmed, selected rats were orally treated with vildagliptin (5 mg/kg/day) or linagliptin (5 mg/kg/day) once daily for 30 days. At the end of the experiment, rats were sacrificed by cervical dislocation and lungs were homogenized for evaluation of the lung values of NF-kB, TNF-α, and IL-1β. Data are presented as mean ± SEM (8). a Significant difference from control group *p* < *0.05*.b Significant difference from STZ group *p* < *0.05*. c Significant difference from STZ + linagliptin(5 mg/kg) group *p* < *0.05*.

Rats treated with STZ exhibited a significant increase in NF-kB, TNF-α, and IL-1β levels by 10-fold, 6.9-fold, and 10-fold, respectively, compared to the normal control group. However, linagliptin (5 mg/kg) treatment led to a significant reduction in NF-kB, TNF-α, and IL-1β levels (*p* < 0.05) by 41%, 34.5%, and 44%, respectively, compared to the STZ group. The STZ + Vildagliptin (5 mg/kg) group also showed a significant decrease in NF-kB, TNF-α, and IL-1β levels (*p* < 0.05) by 19%, 22.5%, and 18.6%, respectively, compared to the STZ group (Fig. 3).

### Effect of the administration of vildagliptin and linagliptin on the expression of the NLRP3 and GSDMD genes in the lungs of diabetic rats

**Fig. 4:**
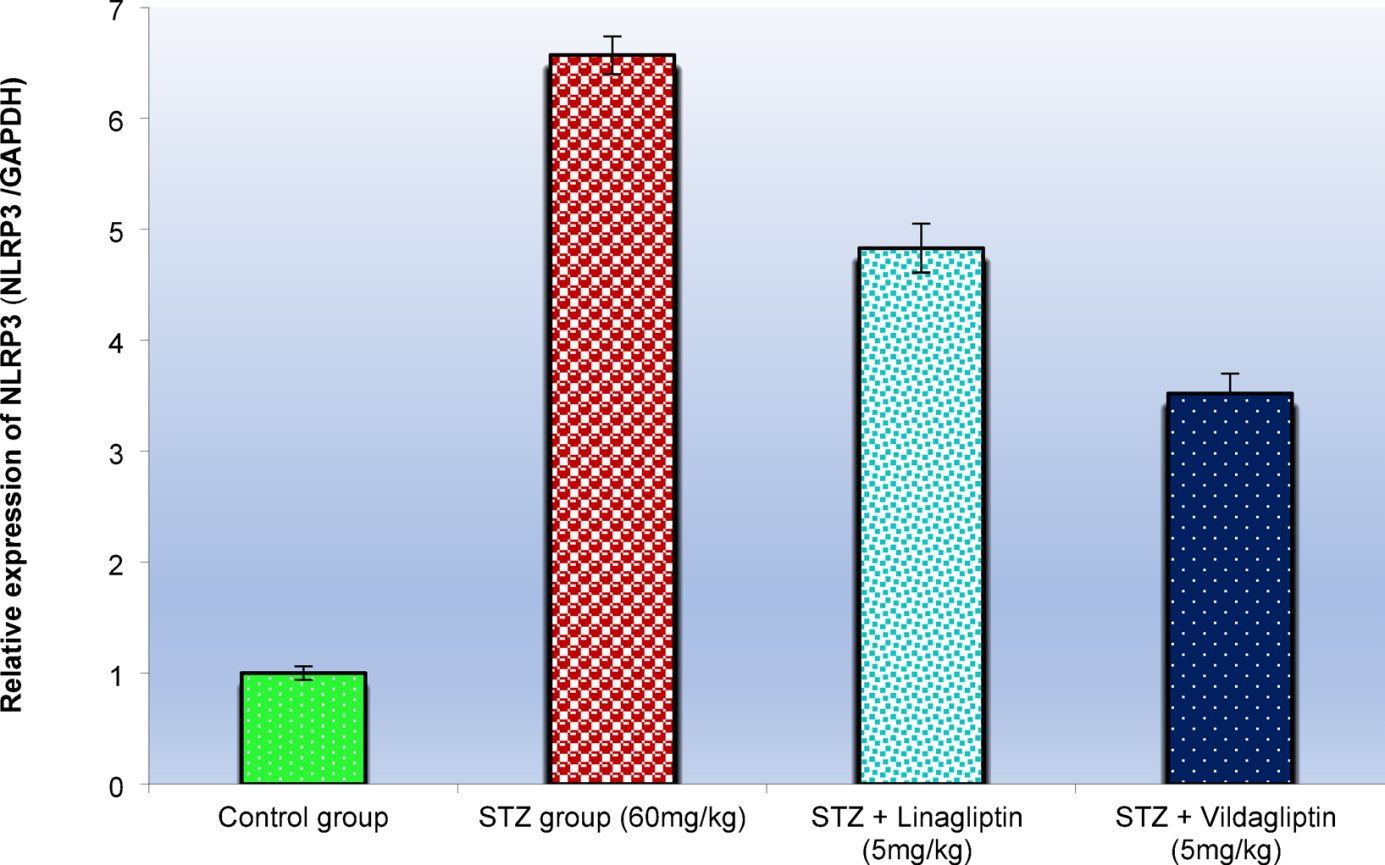
Effect of administration of vildagliptin and linagliptin on the expression alterations of *NLRP3* gene in the lung of diabetic rats. DM was induced by intraperitoneal injection of a single dose of STZ (60 mg/kg b.wt.). After DM was confirmed, selected rats were orally treated with vildagliptin (5 mg/kg/day) or linagliptin (5 mg/kg/day) once daily for 30 days. At the end of the experiment, rats were sacrificed by cervical dislocation and lungs were homogenized for evaluation of the expression alterations of *NLRP3* gene. Data are presented as mean ± SEM (8). a Significant difference from control group *p* < *0.05*.b Significant difference from STZ group *p* < *0.05*. c Significant difference from STZ + linagliptin(5 mg/kg) group *p* < *0.05*.

**Fig. 5:**
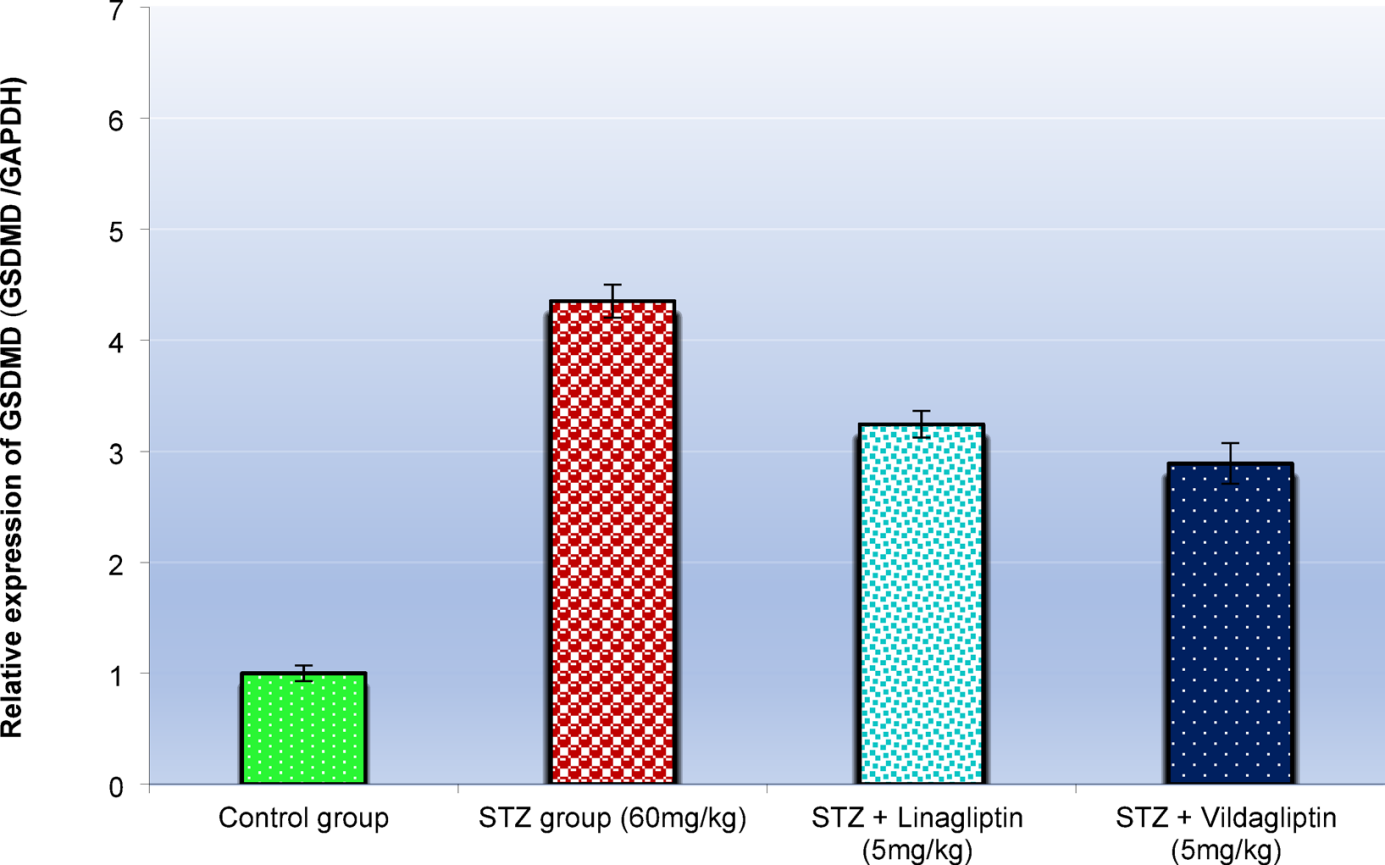
Effect of administration of vildagliptin and linagliptin on the expression alterations of *GSDMD* gene in the lung of diabetic rats. DM was induced by intraperitoneal injection of a single dose of STZ (60 mg/kg b.wt.). After DM was confirmed, selected rats were orally treated with vildagliptin (5 mg/kg/day) or linagliptin (5 mg/kg/day) once daily for 30 days. At the end of the experiment, rats were sacrificed by cervical dislocation and lungs were homogenized for evaluation of the expression alterations of *GSDMD* gene. Data are presented as mean ± SEM (8). a Significant difference from control group *p* < *0.05*.b Significant difference from STZ group *p* < *0.05*. c Significant difference from STZ + linagliptin(5 mg/kg) group *p* < *0.05*.

This study examined the expression of NLRP3 and GSDMD in the lung tissues of control rats, STZ-treated rats, STZ-treated rats with linagliptin, and STZ-treated rats with vildagliptin (Figs. 4 and 5, respectively).

The NLRP3 gene plays a crucial role in the pathophysiology and progression of diabetes mellitus (DM). In comparison to control rats, the expression of NLRP3 was significantly upregulated in the lung tissues of STZ-induced diabetic rats (Fig. 4). However, in STZ-treated rats administered linagliptin, there was a marked reduction in NLRP3 expression (*p* < 0.01), with a 26.5% inhibition rate. Furthermore, STZ-induced rats treated with vildagliptin showed a more substantial decrease in NLRP3 expression (*p* < 0.01), with a 46.4% inhibition rate compared to the diabetic rats. Thus, vildagliptin demonstrated a much greater inhibitory effect on NLRP3 (46.4%) than linagliptin (26.5%) (Fig. 4) (Table 2).

**Table 2:** Effect of administration of vildagliptin and linagliptin on the expression alterations of *NLRP3* gene in the lung of diabetic rats.

Treatment	Mean	SEM	(%) of control	(%) of Disease	(%) Inhibition
Control group	1.0	0.06	100	0.0	0.0
STZ group (60 mg/ kg)	6.57	0.17	657^a^	100 ^a^	0.0^a^
STZ + Linagliptin (5 mg/ kg)	4.83	0.22	483 ^ab^	73.5 ^ab^	26.5 ^ab^
STZ + Vinagliptin (5 mg/ kg)	3.52	0.18	352 ^bc^	53.6 ^bc^	46.4 ^bc^

DM was induced by intraperitoneal injection of a single dose of STZ (60 mg/kg b.wt.). After DM was confirmed, selected rats were orally treated with vildagliptin (5 mg/kg/day) or linagliptin (5 mg/kg/day) once daily for 30 days. At the end of the experiment, rats were sacrificed by cervical dislocation and lungs were homogenized for evaluation of the expression alterations of *NLRP3* gene. Data are presented as mean ± SEM (8). ^a^Significant difference from control group *p* < *0.05*. ^b^Significant difference from STZ group *p* < 0.05. c Significant difference from STZ + linagliptin(5 mg/kg) group *p* < 0.05.

The GSDMD gene plays a critical role in mediating pyroptosis, a form of apoptosis induced by hyperglycemia. The expression levels of GSDMD in the lung tissues of STZ-induced diabetic rats were significantly higher (*p* < 0.01) compared to control rats (Fig. 5). Treatment with linagliptin in STZ-induced rats led to a significant reduction in GSDMD expression (*p* < 0.01), with a 25.5% inhibition rate compared to the diabetic rats. Similarly, vildagliptin treatment significantly (*p* < 0.01) decreased GSDMD expression, with a 33.6% inhibition rate compared to the diabetic rats. Therefore, vildagliptin exhibited a slightly stronger inhibitory effect on GSDMD expression (33.6%) compared to linagliptin (25.5%) (Fig. 5) (Table 3).

**Table 3:** Effect of administration of vildagliptin and linagliptin on the expression alterations of *GSDMD* gene in the lung of diabetic rats.

Treatment	Mean	SEM	(%) of control	(%) of Disease	(%) Inhibition
Control group	1.0	0.07	100	0.0	0.0
STZ group (60 mg/ kg)	4.35	0.15	435 ^a^	100 ^a^	0.0
STZ + Linagliptin (5 mg/ kg)	3.22	0.12	322 ^ab^	74.0 ^ab^	26.0 ^ab^
STZ + Vildagliptin (5 mg/ kg)	2.89	0.18	289 ^bc^	66.4 ^bc^	33.6 ^bc^

DM was induced by intraperitoneal injection of a single dose of STZ (60 mg/kg b.wt.). After DM was confirmed, selected rats were orally treated with linagliptin (5 mg/kg/day) or vildagliptin (5 mg/kg/day) once daily for 30 days. At the end of the experiment, rats were sacrificed by cervical dislocation and lungs were homogenized for evaluation of the expression alterations of *GSDMD* gene. Data are presented as mean ± SEM (8). ^a^Significant difference from control group *p* < 0.05. ^b^Significant difference from STZ group *p* < 0.05. c Significant difference from STZ + linagliptin(5 mg/kg) group *p* < 0.05.

###  Effect of the administration of vildagliptin and linagliptin on histopathological alterations in the lungs of diabetic rats

**Fig. 6:**
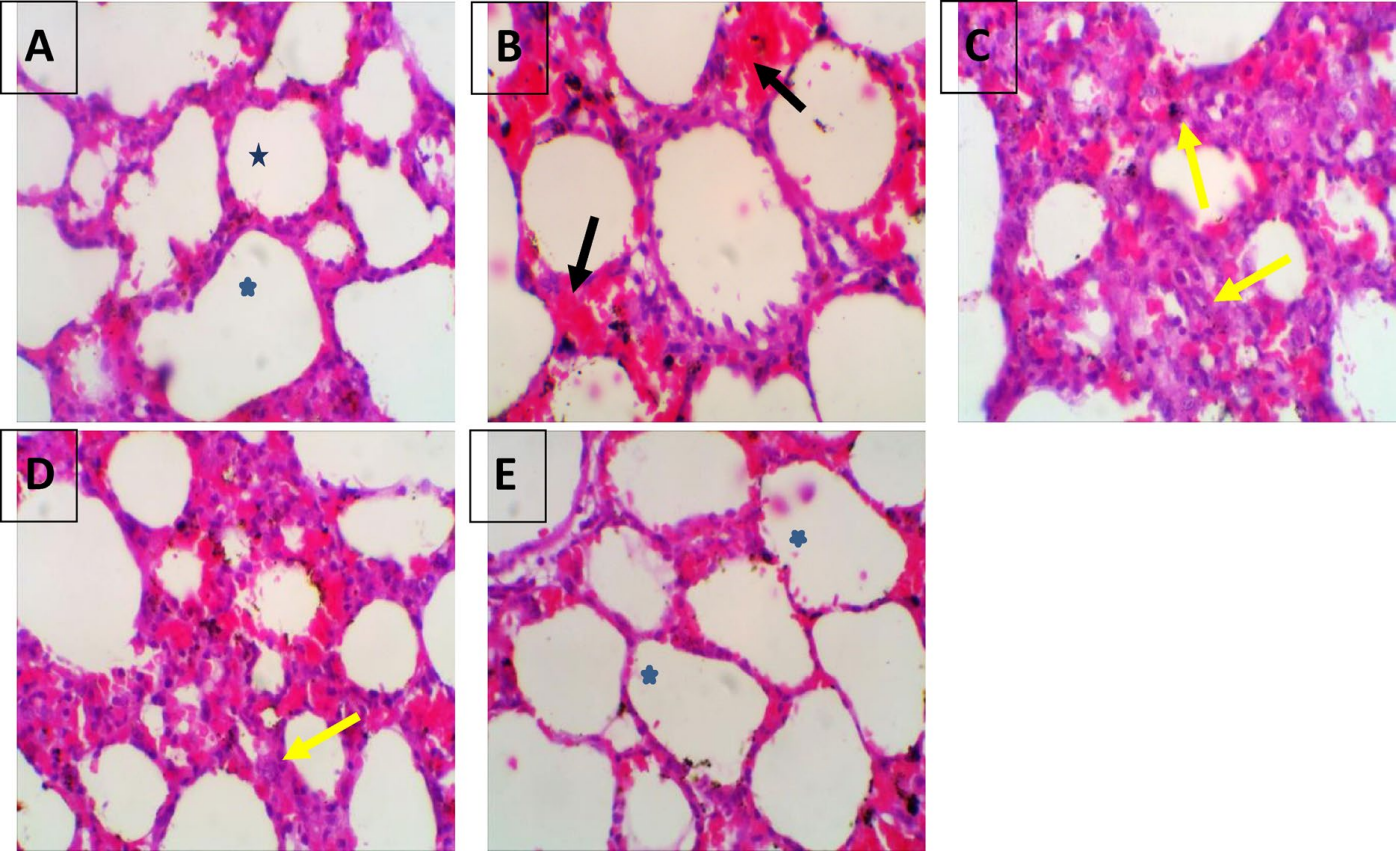
Effect of administration of vildagliptin and linagliptin on the histopathological alterations in the lung of diabetic rats. (**A**) Representative photomicrograph of the control rats showing normal histomorphology architectures and normal pulmonary alveoli (*). (**B**, **C**) STZ group (60 mg/kg) revealed hemorrhages with congested blood capillary (back arrow), and marked obstructions of pulmonary alveoli due to fibroblastic proliferations and chronic inflammatory cells, with severe thickening of alveolar septa (yellow arrow). (**D**) STZ + linagliptin (5 mg/kg) group showed moderate thickening septa (yellow arrow) and diffused alveolar damage. (**E**) STZ + vildagliptin (5 mg/kg) group exhibited nearly normal histomorphology architectures and normal pulmonary alveoli (*). (H&E, ×400).

**Fig. 7:**
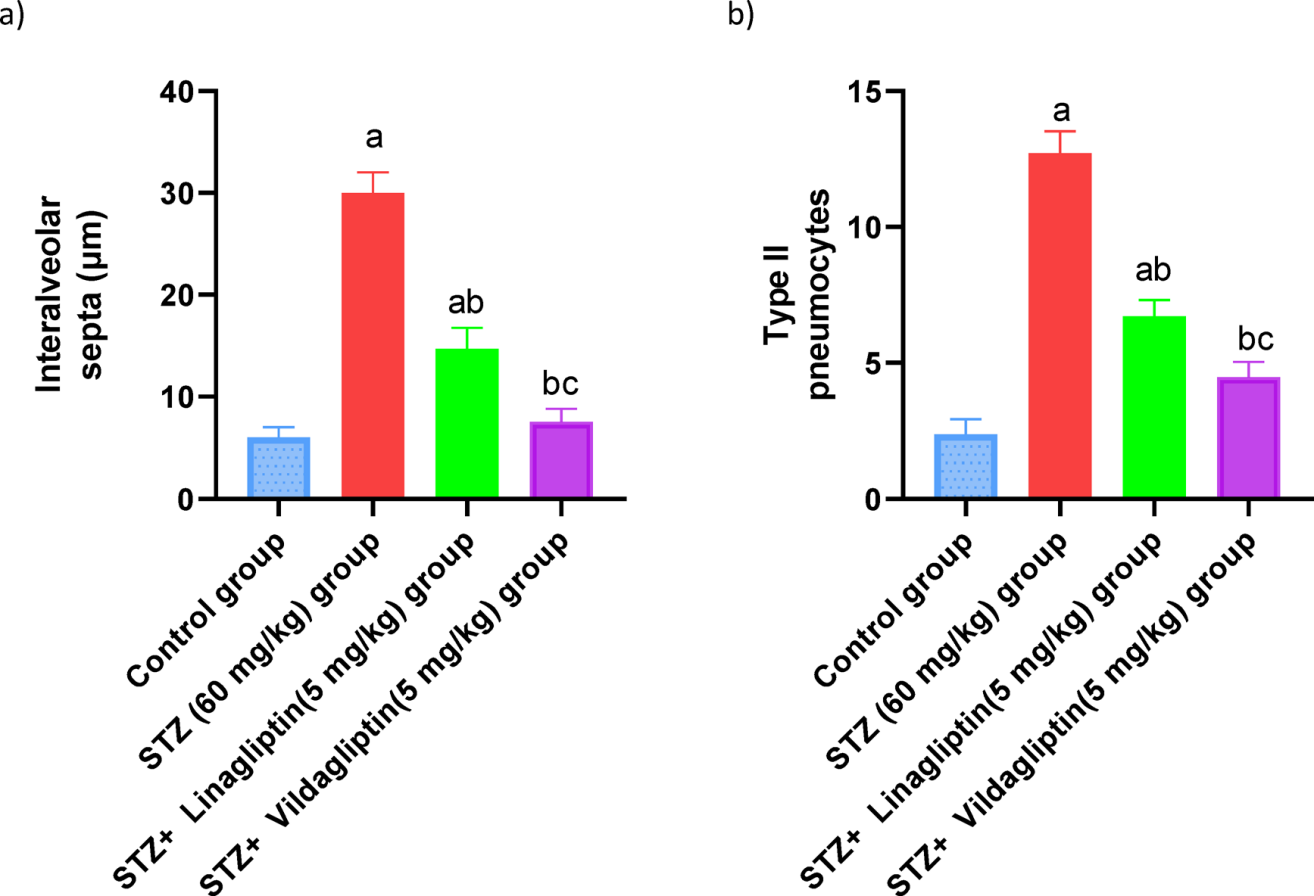
Effect of administration of vildagliptin and linagliptin on the mean values of inter-alveolar septa thickness and type II pneumocytes number in the lung of diabetic rats. DM was induced by intraperitoneal injection of a single dose of STZ (60 mg/kg b.wt.). After DM was confirmed, selected rats were orally treated with vildagliptin (5 mg/kg/day) or linagliptin (5 mg/kg/day) once daily for 30 days. At the end of the experiment, rats were sacrificed by cervical dislocation and lungs were collected promptly and fixed in 10% buffered neutral formalin. The mean values of inter-alveolar septa thickness and type II pneumocytes number were evaluated in the lung of diabetic rats. Data are presented as mean ± SEM (8). a Significant difference from control group *p* < *0.05*.b Significant difference from STZ group *p* < *0.05*. c Significant difference from STZ + linagliptin(5 mg/kg) group *p* < *0.05*.

Histological examination of lung tissues obtained from the normal control rats revealed normal pulmonary alveoli with normal histopathological architecture. The STZ group (60 mg/kg) demonstrated significant blockages of lung alveoli due to fibroblastic proliferations and chronic inflammatory cells, with substantial thickening of alveolar septa. The STZ + linagliptin (5 mg/kg) group showed moderate thickening septa (yellow arrow) and diffused alveolar damage. The STZ + Vildagliptin (5 mg/kg) group exhibited nearly normal histomorphology architectures and normal pulmonary alveoli (Fig. 6). Also, the mean values of inter-alveolar septa thickness and type II pneumocytes were scored in the lung of rats of different experimental groups, where, their counts were significantly elevated in The STZ group (60 mg/kg), as compared with control group. On the other side, the mean values of inter-alveolar septa thickness and type II pneumocytes were significantly reduced in rats treated with linagliptin and restored to their normal values in rats treated with vildagliptin(diabetic rats(Fig. 7).

**Table 4:** Effect of administration of vildagliptin and linagliptin on the expression values of cleaved caspase-3 in the lung of diabetic rats.

Parameter/group	Cleaved caspase-3 (% of positive cells/HPF)
Control group	0.00 ± 0.00
STZ (60 mg/kg) group	3.00 ± 0.11^a^
STZ + Linagliptin(5 mg/kg) group	1.40 ± 0.06^ab^
STZ + Vildagliptin(5 mg/kg) group	0.01467 ± 0.01^bc^

DM was induced by intraperitoneal injection of a single dose of STZ (60 mg/kg b.wt.). After DM was confirmed, selected rats were orally treated with vildagliptin (5 mg/kg/day) or linagliptin (5 mg/kg/day) once daily for 30 days. At the end of the experiment, rats were sacrificed by cervical dislocation and lungs were collected promptly and fixed in 10% buffered neutral formalin. The expression values of cleaved caspase-3 were evaluated. Data are presented as mean ± SEM (8).^a^Significant difference from control group *p* < 0.05. ^b^Significant difference from STZ group *p* < 0.05. ^c^Significant difference from STZ + linagliptin(5 mg/kg) group *p* < 0.05.

###  Effect of administration of vildagliptin and linagliptin on caspase-3 expression in the lungs of diabetic rats

**Fig. 8:**
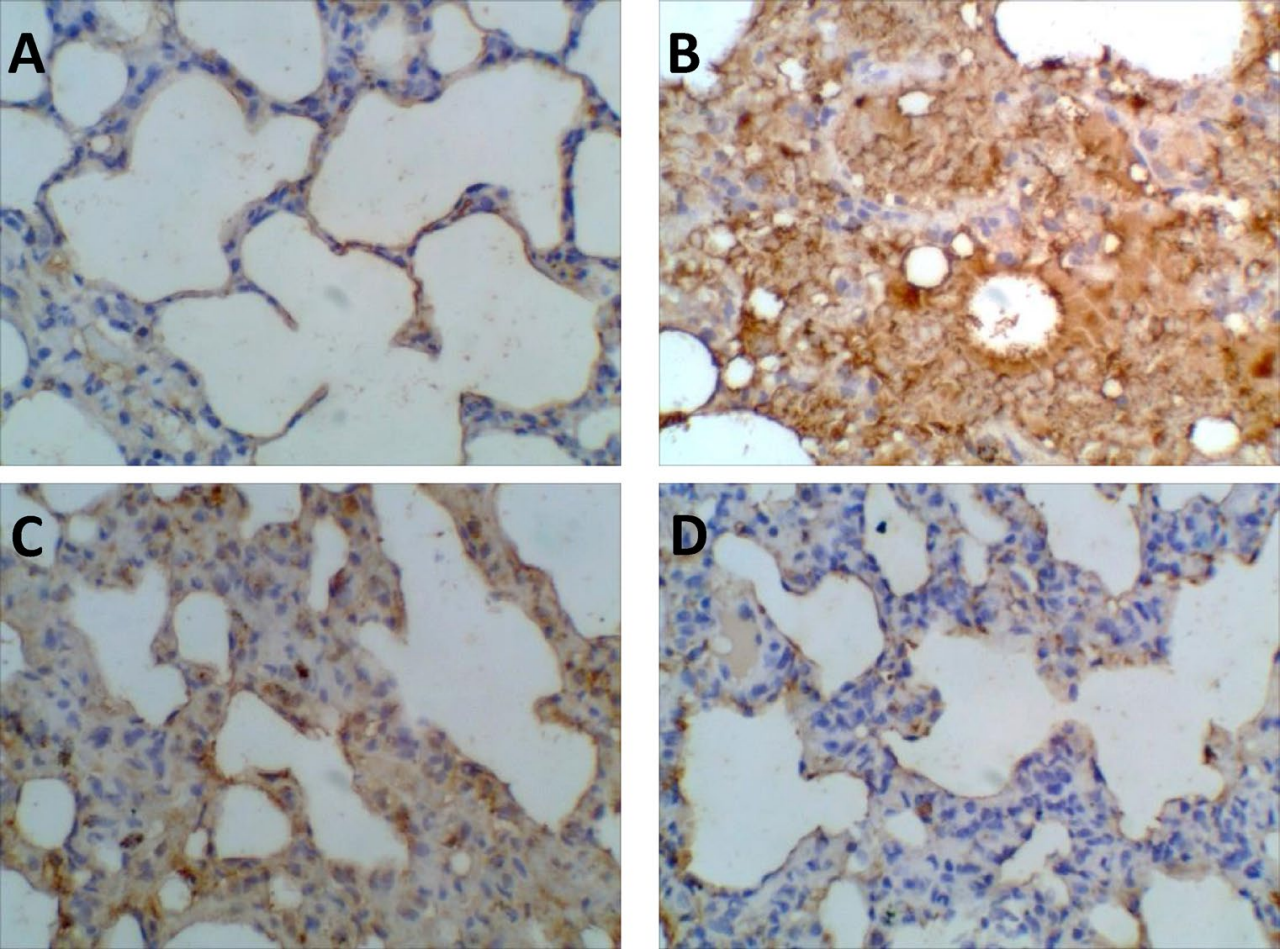
Effect of administration of vildagliptin and linagliptin on the expression of caspase-3 in the lung of diabetic rats. (**A**) Control group exhibited normal lung region with rare expression of caspase-3 immunolabeled cells. (**B**) STZ group (60 mg/kg) showed high characterization by a light brown cytoplasmic caspase-3 staining in and around a necrotic area, while the nucleus remained unstained (blue). (**C**) STZ + linagliptin (5 mg/kg) group revealed marked increase in the number of labeled cells. (**D**) STZ + vildagliptin (5 mg/kg) group exhibited nearly normal control group with very fine expression of caspase 3 immuno – staining (Magnification, ×400).

The normal control group showed no expression of caspase-3 immunolabeled cells. The STZ group (60 mg/kg) exhibited high expression of caspase-3 immunolabeled cells around a necrotic area. The STZ + linagliptin (5 mg/kg) group showed presence of few caspase-3 immunolabeled cells. On the other hand, STZ + vildagliptin (5 mg/kg) group exhibited nearly normal control group with fine expression of caspase 3 immuno-staining (Fig. 8). In addition, the results obtained from (Table 4) revealed that STZ group was associated with significant increases in the expression of Cleaved caspase-3, as compared with control group. On the other side, the expression of Cleaved caspase-3 was significantly reduced in rats treated with linagliptin and restored to its normal values in rats treated with vildagliptin.

## Discussion

Diabetes mellitus (DM) is a persistent global metabolic condition that causes difficulties in multiple organs because of neuronal, microvascular, and macrovascular damage^40^. The complex alveolar‒capillary network of the lungs may be affected by diabetic microvascular damage. However, studies on the consequences of diabetes on lung tissue are lacking 41^43^. DM causes both structural and functional damage to lung tissue 43^45^. The present work designed to elucidate the evidence regarding the lung as the target organ for type 1 DM and the possible mechanism of action of vildagliptin and linagliptin in maintaining lung function and preventing pulmonary damage progression in male Sprague Dawley-rats with STZ-induced DM, especially through the NLRP3 inflammatory pathway.

Diabetes is a complicated condition characterized by hyperglycemia, oxidative stress, and inflammation^46^. Glucotoxicity-related complications are a major cause of mortality in diabetic patients^4^. The current investigation revealed that the rats injected with Streptozotocin (60 mg/kg) showed a significant increase in fasting blood glucose and a decrease in serum insulin levels, confirming the development of DM. These changes in glucose and insulin levels may be attributed to STZ, which is a glucosamine-nitrosourea lipophilic compound. It has the ability to mimics glucose, and to be selectively uptake within the pancreatic beta cells via the low-affinity glucose transporter protein (GLUT-2) due to the existence of the 2-deoxy-d-glucose moiety within its molecular structure^47,48^.

Streptozotocin, produces highly reactive carbonium ions (CH3^+^) that exerts its toxic effects by alkylating cellular DNA, which initiate poly ADP-ribosylation. This process is thought to play a crucial role in the development of diabetes, potentially more so than the DNA damage itself^49^. It diminishes nicotinamide-adenine dinucleotide NAD as well as subsequently ATP stores in pancreatic beta cells, and increased levels of ADP, thereby promoting β-cell degeneration within the islets of Langerhans and leads to the onset of diabetes within three days^50,51^.

Diabetic rats treated with linagliptin presented a significant decrease in blood glucose and an increase in insulin levels. This effect is attributed to linagliptin’s capacity to enhance the glucose-dependent stimulation of insulin secretion and control blood glucose via the inhibition of glucagon secretion and gastric emptying^52^. A previous study highlighted the hypoglycemic effects of linagliptin in a nondiabetic rat model of Parkinson’s disease (PD)^53^. It is important to recognize that the physiological mechanisms operating in non-diabetic models may differ significantly from those in diabetic conditions. Therefore, former study demonstrated that linagliptin significantly improved fasted blood glucose levels, insulin sensitivity and lipid profile as well as reduced inflammatory mediators. Above and beyond its anti-diabetic effect, this study introduced linagliptin as a promising option for preventing the pathological progression of liver fibrosis associated with T2DM.

Interestingly, our findings revealed that treatment with vildagliptin led to a significant decrease in blood glucose and an increase in insulin levels in diabetic rats compared with those in untreated controls. The proposed mechanisms underlying vildagliptin’s glucose-lowering effects primarily involve the enhancement of circulating GLP-1 availability, which in turn improves pancreatic β-cell function. This results in increased insulin secretion in response to food intake, reduced hepatic glucose output through decreased glucagon secretion, decreased insulin resistance, and restoration of pancreatic β-cell mass^54,55^. Moreover, a previous study reported that treatment with vildagliptin partially reversed the decrease in insulin levels in STZ-induced diabetic rats^56^.

Unsaturated fatty acid lipid peroxidation is a prominent sign of heightened oxidative stress as well as consequent oxidative damage, which is commonly associated in chronic diabetes^57^. Lipid peroxidation reduces the fluidity of cell membrane and affects the activity of membrane-bound receptors and enzymes, causing membrane dysfunction^58^. Results of the present study demonstrated a significant increase in malondialdehyde (MDA) levels, while superoxide dismutase (SOD) and glutathione (GSH) levels were notably reduced in the diabetic group following 30 days of STZ treatment, indicating oxidative stress. The increased MDA levels and reduced SOD and GSH concentrations may be a result of STZ-induced hyperglycemia, which exacerbates tissue hitches by promoting lipid peroxidation as well as depleting antioxidant levels^59^. Conversely, diabetic rats treated with linagliptin showed a significant reduction in MDA levels, along with a notable increase in SOD and GSH levels. The findings of this investigation are consistent with earlier studies showing that linagliptin increases antioxidant levels in a rat model of isoproterenol-induced lipid peroxidation^60^. Compared to linagliptin, vildagliptin was superior in restoring redox homeostasis, and previous findings support the significant antioxidant potential of vildagliptin^20,61^.

Furthermore, hyperglycemia induces free oxygen radical release and stimulates the production of proinflammatory cytokines along with the overexpression of NF-κB^62^. NF-κB plays a crucial role in the interaction between oxidative stress and inflammation^63^. As a central regulator of inflammation, NF-κB’s signaling pathway has been shown to contribute to pathological lung inflammation^64^. Notably, NF-κB p65 is the primary subunit sequestered in the cytoplasm and rendered inactive through its connection with inhibitory molecules, notably IκB, where proinflammatory cytokines rapidly degrade IκB, to release the nuclear translocation of NF-κB^65^. TNF-α and Il-1β are critical mediators of NF-κB signaling in the lungs of diabetic rats^66^. A previous report supported the notion that mitochondrial ROS can modulate TNF-α-mediated NF-κB activation, consequently inducing apoptotic cell death^67^. In the current study, administration of linagliptin to STZ-induced diabetic rats resulted in reduced expression of NF-κB and consequently lowered levels of TNF-α and IL-1β. This aligns with previous findings indicating that linagliptin exerts anti-inflammatory effects in amyloid-β cytotoxicity models in human SH-SY5Y neuroblastoma cells^68^. Our investigation found that vildagliptin effectively suppressed NF-κB signaling by reducing oxidative damage and inflammation.

To further understand the effects of linagliptin and vildagliptin on diabetic lungs, we investigated the role of pyroptosis, a kind of inflammatory cell death that contributes to the lung damage^69^. Hyperglycemia generates ROS, which activate the NLRP3 inflammasome, causing diabetic lung injury^70^. The activated NLRP3 proteins polymerize and merge with the apoptosis-associated speck-like protein containing a CARD, causing procaspase-1 to translocate and activate Cas-1^71^. Cas-1 may cleave a full-length GSDMD protein to yield the GSDMD N-terminus. This creates enormous oligomeric pores inside the plasma membrane, permitting the release of Il-1β that initiate the inflammation and cell lysis^72^. A significant increase in the protein expression of NLRP3 and GSDMD in lung tissue was detected, indicating pyroptosis-mediated cell death in the STZ model. The current results regarding pyroptosis and the aetiology of diabetes and its consequences are similar with a study that indicated its function in depression associated with diabetes^73^. While STZ rat group treated with linagliptin showed large decreases in NLRP3 and GSDMD protein levels in the lungs, as well as significant decreases in NLRP3 and GSDMD mRNA expression. These findings are linked to linagliptin’s anti-inflammatory activities by inhibiting the NLRP3 inflammasome. These findings agree with those of a previous study that revealed that linagliptin inhibited the activation of NLRP3-mediated pyroptosis in an MPTP mouse model of PD^74^. Interestingly, STZ-induced rats treated with vildagliptin exhibited a significant anti-inflammatory role in the inhibition of the NLRP3 inflammasome. These findings are in agreement with previous findings in primary human umbilical vascular endothelial cells and revealed that vildagliptin could inhibit high free fatty acid-induced NLRP3 inflammasome activation in endothelial cells^75^. Indeed, the inhibition of pyroptosis is considered a valuable mechanistic insight and a promising strategy for the alleviation of DM complications on the lungs.

Caspase-3, the major controller of apoptosis, is regarded as an important target for detecting lung injury^76^. The STZ-treated rats exhibited an enormous rise in the values of caspase-3 in the lungs that were consistent with the observed histopathological alterations. This study is consistent with earlier research that demonstrated that apoptosis is elevated in endotoxin-induced lung injury^77,78^. Compared with that of linagliptin, the lung values of in STZ- treated rats with vildagliptin were much lower due to the marked antioxidant and anti-inflammatory properties of vildagliptin. In addition, vildagliptin was reported to be a selective competitive reversible inhibitor of DPP-4^79^.

Histopathological investigations of the lung tissue of the STZ rats in the present investigation also revealed hemorrhage with congested blood capillaries, persistent inflammatory cells and severe thickening of the alveolar septa. The histopathological findings in the lung tissue of STZ-induced rats are consistent with prior findings, such as bleeding in the lung tissues induced by STZ, thickening of the alveolar wall, and inflammatory cell infiltration^76^. The vildagliptin group demonstrated considerable improvement in histological abnormalities in comparison with the linagliptin-treated group, with restoration of normal histopathological architecture and pulmonary alveoli. These findings are consistent with a previous study that found vildagliptin could reduce pulmonary fibrosis in lipopolysaccharide-induced lung damage by decreasing the endothelial-mesenchymal transition^77^.

## Conclusions

Lung injury, a diabetic complication, is triggered by pyroptosis, depending on stimulating the NLRP3–GSDMD–Il-1β pathway. Vildagliptin was superior in alleviating diabetes-induced lung dysfunction by inhibiting NLRP3-mediated pyroptosis, oxidative stress, inflammation and could restore the histopathological architecture of the lungs. Thus, vildagliptin could be supposed as a new intervention for reducing diabetic lung consequences.

Diabetic lung injury could be induced through stimulating the NLRP3–GSDMD–il-1β pathway. This pathway is a crucial trigger for the process of pyroptosis. The present study showed that vildagliptin was superior in alleviating diabetes-induced lung dysfunction and in restoring the histopathological architecture of the lungs when compared with linagliptin. These ameliorative effects could be in part mediated via inhibiting NLRP3-mediated pyroptosis, oxidative stress, inflammation. Thus, vildagliptin could be supposed to be a new intervention for reducing diabetic lung consequences. Further studies including clinical investigations are needed to confirm these effects and establish appropriate dosing and safety in humans.

## Data Availability

All data are accessible on request from the corresponding author.
