# Correction: Unravelling the mechanism by which vildagliptin and linagliptin inhibit pyroptosis in lung injury through the NLRP3 inflammatory pathway in type 1 diabetic rats

**DOI:** 10.1038/s41598-025-19552-z

**Published:** 2025-09-15

**Authors:** Ahmed A. Sedik, Nesma M. E. Abo El-Nasr, Wagdy K. B. Khalil, Aliaa E. M. K. El-Mosallamy

**Affiliations:** 1 Pharmacology Department, Medical Research and Clinical Studies Institute, National Research Centre, El-Buhouth St., Dokki, Cairo, 12622 Egypt; 2 Department of Cell Biology, Biotechnology Research Institute, National Research Centre, El-Buhouth St., Dokki, Cairo, 12622 Egypt

Correction to: *Scientific Reports* 10.1038/s41598-025-07204-1, published online 25 June 2025

The original version of this Article contained an error in the name of the author Nesma M.E. Abo El-Nasr, which was incorrectly given as Nesma Esmat. Furthermore, the name of the author Aliaa E.M.K. El-Mosallamy contained errors and was incorrectly given as Aliaa EL-mosallamy.

The Author Contributions section now reads:

Conception: A.A.S.; methodology: A.A.S., A.E.M.K.E.-M., W.K.B.K., N.M.E.A.E.-N.; writing original draft: A.A.S., N.M.E.A.E.-N.; writing and reviewing the final draft: A.A.S., A.E.M.K.E.-M., W.K.B.K., N.M.E.A.E.-N. All the authors have read and approved the manuscript.

The original Article has been corrected.

